# Polyphenols-Rich Fruit (*Euterpe edulis* Mart.) Prevents Peripheral Inflammatory Pathway Activation by the Short-Term High-Fat Diet

**DOI:** 10.3390/molecules24091655

**Published:** 2019-04-27

**Authors:** Aline Boveto Santamarina, Giovana Jamar, Laís Vales Mennitti, Daniel Araki Ribeiro, Caroline Margonato Cardoso, Veridiana Vera de Rosso, Lila Missae Oyama, Luciana Pellegrini Pisani

**Affiliations:** 1 Programa de Pós-Graduação Interdisciplinar em Ciências da Saúde, Universidade Federal de São Paulo, Santos 11015-020, Brazil; alinesantamarina@gmail.com (A.B.S.); gi.jamar@gmail.com (G.J.); laisvmennitti@hotmail.com (L.V.M.); camargonato@gmail.com (C.M.C.); 2 Departamento de Biociências, Universidade Federal de São Paulo, Santos 11015-020, Brazil; daribeiro@unifesp.br (D.A.R.); veriderosso@yahoo.com (V.V.d.R.); 3 Departamento de Fisiologia, Universidade Federal de São Paulo, São Paulo 04023-062, Brazil; lmoyama@gmail.com; 4 Laboratório de Nutrição e Fisiologia Endócrina (LaNFE), Departamento de Biociências, Instituto de Saúde e Sociedade, Universidade Federal de São Paulo, Rua Silva Jardim, 136, Térreo, Vila Mathias, Santos, São Paulo 11015-020, Brazil

**Keywords:** inflammation, short-term high-fat diet, juçara, nutraceutical food, liver, adipose tissue

## Abstract

Juçara berry is a potential inflammatory modulator, rich in dietary fiber, fatty acids, and anthocyanins. Considering this, we evaluated the high-fat diet (HFD) intake supplemented with different doses of freeze-dried juçara pulp on the TLR4 pathway. Twenty-seven male Wistar rats with ad libitum access to food and water were divided into four experimental groups: control standard chow group (C); high-fat diet control group (HFC); high-fat diet juçara 0.25% group (HFJ0.25%); and high-fat diet juçara 0.5% group (HFJ0.5%). The inflammatory parameters were analyzed by ELISA and Western blotting in liver and retroperitoneal adipose tissue (RET). The HFJ0.25% group had the energy intake, aspartate transaminase (AST) levels, and liver triacylglycerol accumulation reduced; also, the tumor necrosis factor α (TNF-α) and TNF receptor-associated factor 6 (TRAF6) expression in RET were reduced. However, there were no changes in other protein expressions in liver and adipose tissue. Adiposity and pNFκBp50 had a positive correlation in HFC and HFJ0.5%, but not in the C group and HFJ0.25%. The necrosis hepatic score did not change with treatment; however, the serum (AST) levels and the hepatic triacylglycerol were increased in HFC and HFJ0.5%. These results demonstrated that one week of HFD intake triggered pro-inflammatory mechanisms and liver injury. Additionally, 0.25% juçara prevented inflammatory pathway activation, body weight gain, and liver damage

## 1. Introduction

Western diet patterns are spread worldwide and cause the establishment of several non-communicable metabolic diseases. However, many deleterious mechanisms related to high-fat diet exposure are activated even before weight gaining reaches significant levels [1].

There is extensive literature regarding the features of long-term high-fat diet consumption, as well as its consequences for health [2]. At the same time, concerning the effects of short-term high-fat diet consumption, the literature becomes scarcer. Reports are mainly related to saturated fatty acids’ (SFA) damaging action in the central nervous system and satiety control; however, the peripheral metabolic effect is slightly explored [3]. Turner et al. (2013) demonstrated a chronology of activation in inflammatory mechanisms, insulin resistance, and non-alcoholic fatty liver diseases (NAFLD), suggesting that before clinical signs, significant physiological changes may occur [4].

One of the mechanisms involved in the onset of low-grade inflammation in vitro and in vivo models of obesity is mediated by toll-like receptor 4 (TLR4) stimulated by dietary SFA. The TLR4 recognizes lipid ligands and plays an important role in non-infectious inflammatory diseases such as insulin resistance, obesity, and NAFLD. Moreover, polyunsaturated fatty acids (PUFA) ω-3 and ω-6 inhibit the inflammatory response mediated by TLR4 [5,6].

Metabolic disease control using bioactive food compounds has received attention from the scientific community and clinical practice [7]. One Brazilian native fruit with remarkable high nutritional value is juçara fruit. It contains significant amounts of dietary fiber, monounsaturated fatty acids (MUFA), and PUFA. It also has high levels of flavonoids, such as anthocyanins [8]. Therefore, it could contribute to preventing obesity, oxidative stress, and metabolic syndrome, possibly through an anti-inflammatory effect [9,10].

Although it is well known that there is an established chronic subclinical inflammatory state in obesity, only a few studies address the effect of the short-term high-fat diet and food bioactive compounds’ intake. Considering the lack of studies in this field, we aim to evaluate the effectiveness of different juçara doses to prevent the deleterious effects of the short-term high-fat diet intake in the inflammatory markers and ectopic fatty liver accumulation.

## 2. Results

### 2.1. Diet Intake, Body Weight, and Tissue Weight

**Figure 1:**
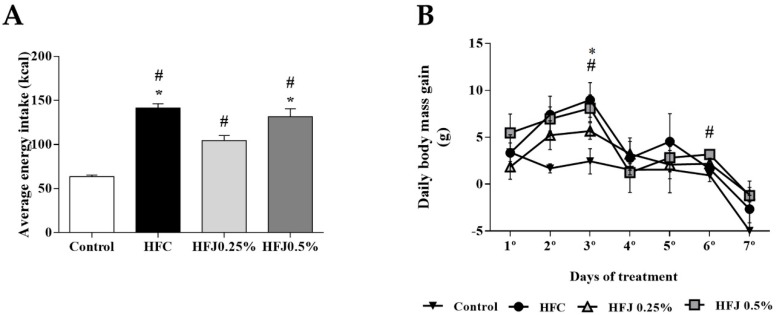
(**A**) Average energy intake (kcal). * *p* < 0.05 compared with the high-fat juçara 0.25% group (HFJ0.25%); # *p* < 0.05 compared with control diet by two-way ANOVA followed by the Bonferroni post-hoc test. (**B**) Daily body weight gain during the experimental period of seven days. The comparisons were performed by ANOVA for repeated measure and the Bonferroni post hoc test. # *p* < 0.05 in HFJ0.5% group compared with the control diet group; * *p* <0.05 in high-fat diet control (HFC) group compared with the control diet group. Control standard chow (C) (*n* = 6); high-fat diet control (HFC) (*n* = 7); high-fat diet juçara 0.25% (HFJ0.25%) (*n* = 7); and high-fat diet juçara 0.5% (HFJ0.5%) (*n* = 7).

The average of energy intake (Kcal) along the treatment was higher in the high-fat diet control (HFC) group compared to the control (C) group (*p* < 0.001) and HFJ0.25% (*p* = 0.0014). The high-fat diet juçara 0.5% (HFJ0.5%) group had a higher energy intake compared to the C group (*p* < 0.001), and HFJ0.25% (*p* = 0.029). The HFJ0.25% group showed higher energy intake than the C group (*p* = 0.0013). The daily body weight gain was accessed day by day during the experiment. At the third day of treatment, the HFC group and HFJ0.5% had a greater body weight gain compared to the C group (*p* = 0.006 and 0.016, respectively). After six days of diet exposure, the HFJ0.5% group demonstrated an increased body weight gain comparing to the C group (*p* = 0.012). However, the other measures did not demonstrate differences among the groups regarding body weight gain (Figure 1).

Retroperitoneal and epididymal white adipose tissue (RET and EPI, respectively) absolute weights were increased in the HFC compared to the C group. Regarding the different adipose tissues evaluated, both juçara-supplemented groups (HFJ0.25% and HFJ0.5%) maintained a similarity between other groups or themselves. Liver and mesenteric white adipose tissue (MES) weight did not change among the experimental groups. The sum of white adipose tissues’ weight depots (ΣWAT) was significantly increased in the HFC group compared to the control (Table 1).

### 2.2. Hepatic Ectopic Fat Accumulation 

**Figure 2:**
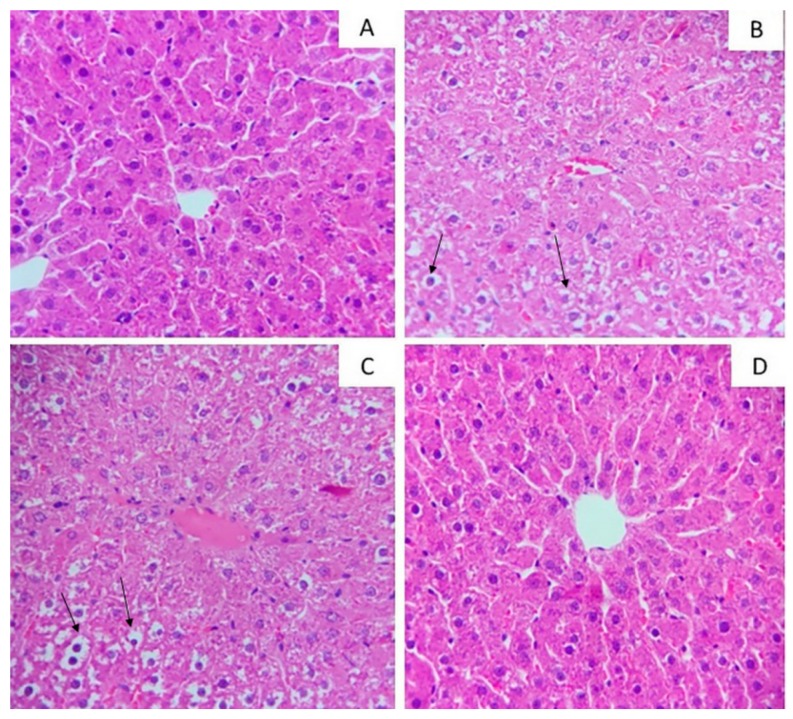
Photomicrographs of hepatic tissue stained by hematoxylin and eosin with a magnification of 40×. (**A**) Control standard chow (control); (**B**) high-fat diet control group (HFC); (**C**) high-fat diet juçara 0.5% group (HFJ0.5%); (**D**) high-fat diet juçara 0.25% group (HFJ0.25%). The arrows (↑) indicate the lipid droplets.

Liver histological analyses performed in hematoxylin and eosin illustrated the morphological differences among the groups (Figure 2). The necrosis histopathological score did not statistically change with the experimental treatment (Table 2). However, in the groups exposed to high-fat diet juçara 0.5%, microsteatosis in liver parenchyma was evidenced.

The hepatic triacylglycerol content in HFC and HFJ0.5% groups was increased in comparison of the C group (*p* = 0.0055 and 0.0275, respectively). Even though, HFJ0.25% did not differ from the control group, as shown in Table 3. 

Accessing the serum levels of hepatic enzymes—aspartate transaminase (AST) and alanine transaminase (ALT)—there were no differences among the groups. However, the AST level was higher in the HFC and HFJ0.5% groups compared to the C group (*p* = 0.044 and 0.041, respectively). The level of AST in HFJ0.25% was similar to the C group (Table 3). 

### 2.3. Cytokines Concentration

**Figure 3:**
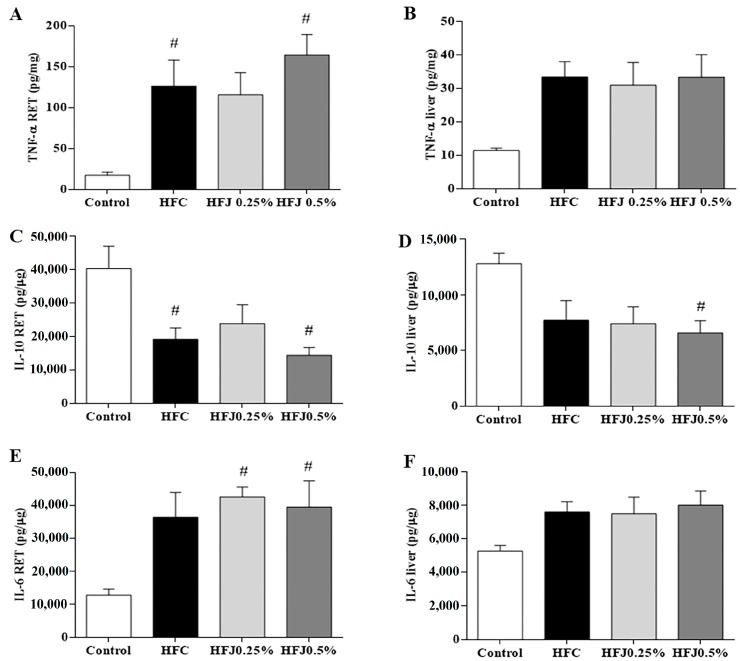
Cytokine levels: (**A**) TNF-α in RET; (**B**) TNF-α in liver; (**C**) IL-10 in RET; (**D**) IL-10 in liver; (**E**) IL-6 in RET; (**F**) IL-6 in liver. # *p* < 0.05 compared with the control diet by two-way ANOVA followed by the Bonferroni post-hoc test. Control standard chow (control) (*n* = 6); high-fat diet control (HFC) (*n* = 7); high-fat diet juçara 0.25% (HFJ0.25%) (*n* = 7); and high-fat diet juçara 0.5% (HFJ0.5%) (*n* = 7).

We observed that IL-6 increased in RET of the HFJ0.25% (*p* = 0.020) and HFJ0.5% (*p* = 0.035) groups compared to the C group. The TNF-α level in RET was increased in the HFC and HFJ0.5% groups compared to the C group (*p* = 0.033 and *p* = 0.003, respectively). In contrast, the HFJ0.25% group was similar to the C group. The IL-10 level was reduced in HFC (*p* = 0.032) and HFJ0.5% (*p* = 0.009) compared to the C group in RET (Figure 3A,C,E). Liver pro-inflammatory cytokines (IL-6 and TNF-α) did not differ among the groups, however, the anti-inflammatory cytokine (IL-10) was lower in the HFJ0.5% group than in the control (*p* = 0.05) group (Figure 3B,D,F).

### 2.4. NFκB Pathway Protein Expression

**Figure 4:**
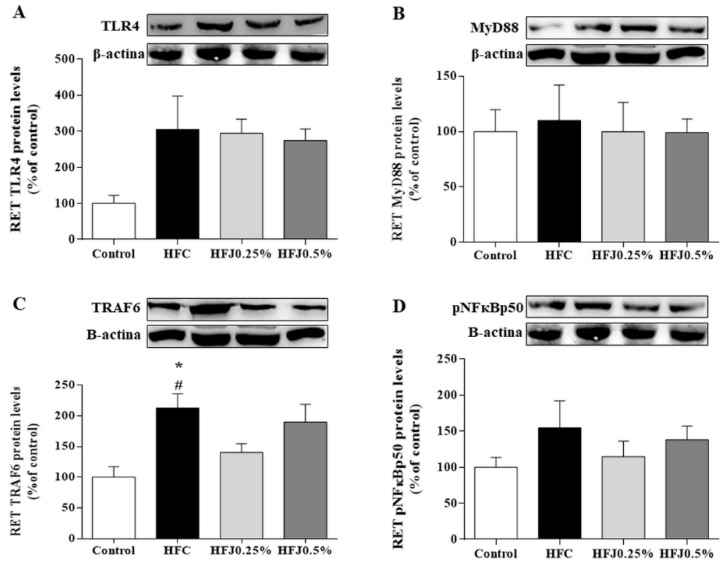
Protein expression of the NFκB pathway in the RET: (**A**) TLR4; (**B**) MYD88; (**C**) TRAF6; and (**D**) pNFκBp50. The housekeeping used was the β-actin expression. * *p* < 0.05 compared with the high-fat juçara 0.25% group (HFJ0.25%); # *p* < 0.05 compared with the control diet. Two-way ANOVA followed by the Bonferroni post-hoc test were performed as the statistical analyses. Control standard chow (control) (*n* = 6); high-fat diet control (HFC) (*n* = 7); high-fat diet juçara 0.25% (HFJ0.25%) (*n* = 7); and high-fat diet juçara 0.5% (HFJ0.5%) (*n* = 7).

The inflammatory TLR4 pathway in RET was evaluated. TNF receptor-associated factor 6 (TRAF6) protein expression was significantly reduced in the HFJ0.25% (*p* = 0.048) as in the C group (*p* = 0.043) compared to the HFC group (Figure 4A–D).

**Figure 5:**
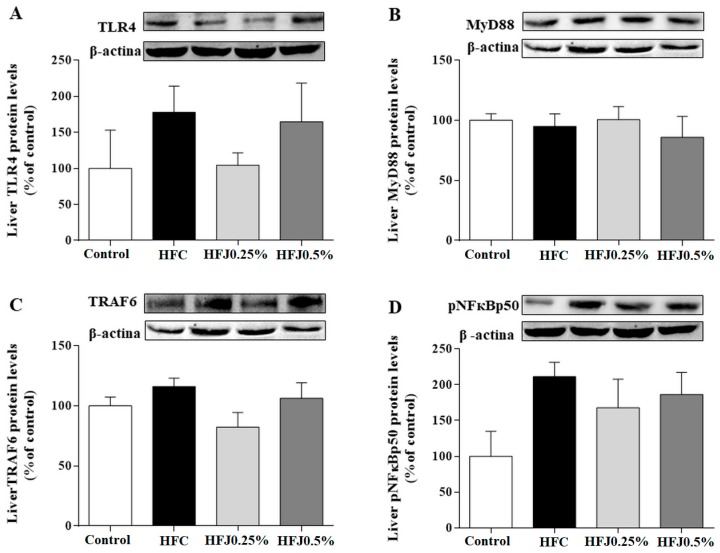
Protein expression of the NFκB pathway in liver: (**A**) TLR4; (**B**) MYD88; (**C**) TRAF6; and (**D**) pNFκBp50. The housekeeping was the β-actin expression. Two-way ANOVA followed by the Bonferroni post-hoc test were performed as the statistical analyses. Control standard chow (control) (*n* = 6); high-fat diet control (HFC) (*n* = 7); high-fat diet juçara 0.25% (HFJ0.25%) (*n* = 7); and high-fat diet juçara 0.5% (HFJ0.5%) (*n* = 7).

There were no differences in protein expression among TLR4 membrane receptors and their cellular signaling intermediates, such as myeloid differentiation primary response 88 (MYD88) and nuclear factor kappa-B p50 (pNFκBp50) in RET and liver. Furthermore, we did not find changes in the protein expression of the TLR4 pathway in liver (Figure 5A–D).

**Figure 6:**
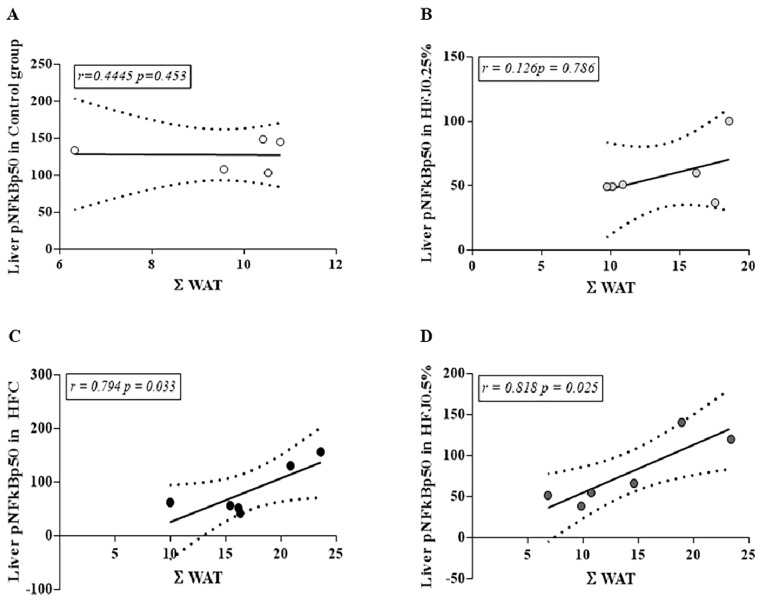
Correlation between ΣWAT and pNFκBp50 in liver for different experimental groups: (**A**) Control standard chow group (control); (**B**) high-fat diet juçara 0.25% (HFJ0.25%); (**C**) high-fat diet control group (HFC); (**D**) high-fat diet juçara 0.5% (HFJ0.5%). The Pearson correlation coefficient was performed considering the level of significance as *p* < 0.05 and the Pearson’s *r-*value *>*0.7 for a strong correlation.

Analyzing the correlation between visceral adiposity (ΣWAT) and phosphorylation of hepatic NFκBp50, it is possible to note a strong positive correlation in the HFC group (*r* = 0.794; *p* = 0.033) and HFJ0.5% (*r* = 0.818; *p* = 0.025). On the other hand, the C and HFJ0.25% groups did not demonstrate this relation (Figure 6), demonstrating the higher effectiveness of the lower dose, in agreement with the results presented.

## 3. Discussion

The results demonstrated the influence of juçara supplementation—a fruit rich in anthocyanin and unsaturated fatty acids (MUFAs and PUFAs)—in energy intake, in adipose tissue and hepatic ectopic fat accumulation, as well as in the inflammatory profile. Regarding the TLR4 pathway, the main benefits are related to the consumption of the lower dose of the pulp.

The higher energy intake observed in the HFD chow groups compared to the control group evidence changes in eating habits due to the high energy density provided by the diet. The HFD can modulate the central regulation of appetite and satiation through intricate ways involving neuroendocrine signaling from peripheral tissues. A recent short-term HFD model study demonstrated that serum ghrelin level is high in postprandial conditions, suggesting that in HFD, the ghrelin orexigenic signal remains activated in the hypothalamus, leading to exacerbated caloric intake [11]. It is noteworthy that the HFJ0.25% group had a smaller caloric intake compared to the other HFD groups. This result can be justified by the protective hypothalamic effect promoted by the juçara supplementation due to its rich composition against the deleterious effects of HFD [12,13].

The increased adiposity (ΣWAT, RET, and EPI) in HFC indicated that our HFD model was efficient at inducing visceral fat weight gain even in a short-term diet treatment [13,14]. High-fat diet consumption is related to the pro-inflammatory mechanism stimulus, leading to chronic subclinical inflammation, promoting metabolic diseases’ installation [15]. According to our findings, previous reports showed that for short-term periods, the high-fat diet is not able to promote relevant changes in body weight gain. However, the adipose pads were slightly increased, as well as the concentration of inflammatory cytokines (IL-6 and TNF-α), similar to our results [14,16,17]. These findings enforce that high-fat diet leads to deleterious pro-inflammatory pathway activation. Both groups subjected to juçara supplementation did not statistically differ from the lean group (C), in adipose depots’ evaluated mass and ΣWAT. This suggests that juçara has a protective role, preventing high-fat diet’s deleterious consequences in visceral adiposity gain. This can be corroborated by other short-term protocols of bioactive compounds’ supplementation, which exert a metabolic protective role in experimental and clinical models [18,19,20,21]. Juçara has beneficial effects previously reported when associated with a high-fat diet, and it may be attributed to the nutritional components of juçara pulp [12,13,22].

The liver histological image illustrates, and TAG content confirms, that the lower dose of juçara (HFJ0.25%) had less injury related to high-fat diet consumption than the HFC and HFJ0.5% groups, remaining at levels similar to those observed in the control group. Furthermore, the ectopic TAG accumulation represents a trigger of NAFLD mechanisms. As a consequence, juçara consumption leads to a protective effect of the regulatory mechanisms for liver. AST and ALT levels are important liver injury markers. These enzymes can be found in hepatocytes cytoplasm and catalyze amino group transferences in the citric acid cycle. Hepatic injury is commonly related to AST and ALT release into the extracellular compartment with subsequent increase in serum. Our results demonstrate that liver integrity was preserved in the HFJ0.25% group, corroborating the reduced liver injury score observed in this group. These findings together indicate that juçara supplementation promoted a hepatic protective effect at the lower dose. Our hypothesis is supported by supplementations of anthocyanins, which protect against NAFLD in long-term high-fat feeding [23,24].

It is interesting to observe that a 0.5% juçara dose did not demonstrate greater efficiency in the modulation of cytokines involved in inflammatory profile and maintained the same level of HFC with ambiguous results in RET. IL-10 reduction in liver, only in the HFJ0.5% group, is an indicator of juçara dose excess. We postulated that high doses might exert a pro-oxidative and negative effect due to high phenolic compound and fatty acid amounts. Lecci et al. (2014) [25] corroborate this hypothesis since non-toxic polyphenol doses activated pro-apoptotic mechanisms in vitro. Despite being a physiological polyphenol and anthocyanin dose, it could exert a cytotoxic role. An in vitro adipocyte study showed that some types of polyphenols act in a pro-oxidant manner, stimulating the production of the pro-inflammatory cytokines, such as TNF-α [26]. Moreover, PUFA and MUFA have deleterious effects when consumed in substantial amounts. This highlights the importance of the fatty acid quality and polyphenol amount offered in the metabolic and inflammatory outcomes [27,28].

It has been shown that the mainly pro-inflammatory cytokines’ source was the adipose tissue compared to liver in obesity and NAFLD. This enforces that peripheral tissues, such as adipose tissue, can affect disease processes in target organs [29]. This matches our results; for the effects in short-term, the adipose tissue showed greater susceptibility to the intervention for inflammatory response compared to liver.

An extensive literature review demonstrates a strong relationship between the TLR4 pathway and high-fat diet consumption in metabolic diseases [1,6]. Turner et al. (2013) have observed that changes from high-fat diet consumption occur progressively among different tissues. Although inflammatory markers in adipose tissue can be noticed after 1–3 weeks of a high-fat diet, hepatic effects are pronounced only after chronic consumption [4]. We observed no changes in TLR4, MYD88, or phosphorylated NFκB expression in RET. However, TRAF6 in RET with a dose of 0.25% was similar to the C group and reduced in comparison to the HFC group. This indicates a possible TLR4 pathway modulation mechanism, corroborating the changes observed in the cytokines’ concentration in adipose tissue. Nevertheless, the diet duration was not enough to install the classic pro-inflammatory framework in liver. Analyzing the correlation between pNFκBp50 and the adiposity index (ΣWAT), the strong correlation between them in the HFC group and HFJ0.5% is noteworthy, but not in C and HFJ0.25%. This exposes two results. Firstly, it demonstrates that adiposity is strongly related to the NFκBp50 phosphorylation, and it could be attributed to high-fat diet intake and its deleterious properties [30]. Secondly, the 0.25% juçara dose was similar to the control group, showing that juçara was able to prevent the deleterious high-fat diet effects, without a correlation between this variable. NFκBp50 is an important nuclear transcription factor, which is activated by kappa B inhibitor degradation mediated by inflammatory signaling TLR4 pathways. NFκBp50’s main function is to induce cytokines’ production, such as TNF-α, playing a role in an inflammatory pathway [15]. The high-fat diet intake, as well as increased adiposity induce the formation of reactive oxygen species (ROS) due to the H_2_O_2_ production in the mitochondria and the peroxisomal oxidation in lipid metabolism; which may cause TLR4 activation [31]. Several bioactive compounds, such as anthocyanins, have been related to inhibiting ROS formation and, consequently, the TLR4 pathway activation [32]. The most relevant finding indicates that juçara effectiveness prevents the pro-inflammatory status installation. Zero-point-two-five percent of juçara associated with a high-fat diet inhibited the inflammatory response, reducing NFκBp50 phosphorylation, despite the high-fat diet’s evident pro-inflammatory stimulus.

## 4. Materials and Methods 

### 4.1. Short-Term High-Fat Diet

A total of 27 outbred male Wistar rats of 90-day-old were used. After one week of acclimatization, the animals were randomly divided into four experimental groups: control standard chow (C) (*n* = 6); high-fat diet control (HFC) (*n* = 7); high-fat diet juçara 0.25% (HFJ0.25%) (*n* = 7); and high-fat diet juçara 0.5% (HFJ0.5%) (*n* = 7). They received the respective diets for the acute experimental period of 7 days.

The C group was fed with standard rat commercial chow, and the composition of the experimental diets was adapted from Dornellas et al. [33] and is described in Table 4. The animals were weighed, and diet consumption was measured every day during the experimental period.

### 4.2. Freeze-Dried Juçara Pulp Powder

Juçara pulp was obtained from the Agro-ecological Juçara Project/Instituto de Permacultura e Ecovilas da Mata Atlântica (Ubatuba, São Paulo, Brazil) and freeze-dried into a powder. The juçara pulp powder was analyzed to confirm the pulp fruit chemo profile. For fiber content, anthocyanins’ and phenolic compounds’ analysis, the HPLC-PDA-MS/MS technique was performed as by Azevedo da Silva et al. (2014), and for the fatty acid analysis profile, the GC-FID-MS/MS technique was used. The results obtained in this characterization are shown in Table 5, expressed as a dry basis. The main anthocyanins and phenolic compounds detected in juçara pulp powder had already been detected in the frozen pulp [8] and are summarized in Table 5.

Test doses were chosen based on juçara pulp anthocyanin levels for physiological consumption. In this study, we proposed two different doses: 0.25% and 0.5% of juçara freeze-dried pulp diet supplementation based on previous studies with anthocyanin supplementation. It has been described that intake from 100.5–350.0 mg per day of anthocyanin is safe and improves the lipid profile and inflammatory responses [24,34].

The average daily anthocyanin intake in the upper dose (0.5%) was equivalent to 6 mg/rat/day, corresponding to a physiological and non-pharmacological dose [35]. The proportional dose for human consumption of juçara was calculated by the allometric factors proposed by the FDA in 2005 [36], considering an adult of 70 kg. The 0.5% dose corresponded to 3.3 mg of anthocyanins/kg/day, which could be obtained by consuming 100 g of fresh juçara pulp or 10 g of the same lyophilized per day. The dose of 0.25% represents 50 g of fresh juçara pulp or 5 g of the freeze-dried juçara pulp per day.

### 4.3. Animal Procedures

Animal procedures were approved by the Experimental Research Committee of the Universidade Federal de São Paulo (No. 5252010715), following the standards of the Brazilian Guidelines for Care and Use of Animals for Scientific Purposes and Teaching by the National Council of Animal Experimentation Control in 2013. The animals were maintained in collective cages with controlled temperature (23 ± 2 °C), humidity (60 ± 5%) and lighting (12-h light/dark) and received ad libitum water and diet.

At the end of the treatment, the animals were anesthetized with ketamine (80 mg/kg) and xylazine (10 mg/kg) and euthanized by beheading in the morning, after fasting for 12 h, between 8:00 and 10:00 Tissue samples were collected for analyses, immediately weighed, and stored at −80 °C.

### 4.4. Histopathological Analysis

Right lobe liver sections were collected and fixed in 10% buffered formalin for 24 h. The samples were processed and paraffin embedded. The histological sections of 4–5 µM were stained using hematoxylin and eosin for histopathological analysis. 

The classification was based on score levels, considering the area of necrosis and changes such as eosinophilia, the presence of intracytoplasmic vacuoles, congestive vessels, and loss of structure established by Silva et al. [37] and described in Table 6.

### 4.5. Hepatic Triacylglycerol Analysis.

The liver tissue samples were homogenized and centrifuged for 5 minutes at 655.1× *g* at room temperature. The lipids extraction was performed as by Folch et al [38], and the total lipid extracts were evaporated under a nitrogen (N_2_) atmosphere and emulsified in a 3% solution of Triton X-100 (Sigma-Aldrich, St. Louis, MO, USA). The TAG was quantified by the colorimetric method, using the Labtest^®^ (Lagoa Santa, Minas Gerais, Brazil) commercial kit.

### 4.6. Hepatic Enzymes in Serum

The hepatic enzymes aspartate transaminase (AST) and alanine transaminase (ALT) in serum were measured by the colorimetric kinetic method using commercial kits (Labtest^®^, Lagoa Santa, Minas Gerais, Brazil).

### 4.7. Tissue TNF-α, IL-6, and IL-10 Concentrations

The liver and RET protein extracts were used in the commercial kits of ELISA (Duo Set ELISA, R&D Systems, Minneapolis, MN) to measure the concentrations of tumor necrosis factor-α (TNF-α), interleukin-6 (IL-6), and interleukin-10 (IL-10) following the manufacturer’s recommendations.

### 4.8. Western Blotting Analyses

Liver and retroperitoneal adipose tissue (RET) protein samples were extracted and measured by the Bradford method. Protein samples were separated by electrophoresis on 10% SDS-polyacrylamide gels and transferred to nitrocellulose membranes. The membranes were blocked with 1% bovine serum albumin and incubated overnight with the following primary antibodies: pNFκBp50 (sc-101744) (Santa Cruz, CA, USA). The TLR4 (ab22048), MYD88 (ab2064), TRAF6 (ab33915), and β-actin (ab6276) were from ABCAM (Cambridge, U.K.).

UVITec (Cambridge, U.K.) and ECL reagent (Bio-Rad Laboratories, Hercules, CA, USA) were used to obtain the bands. Samples were quantified by ImageJ software (ImageJ, National Institute of Health, Maryland, MD, USA). The target proteins levels were normalized to β-actin expression.

### 4.9. Statistical Analyses

Grubb’s test was performed to remove significant outliers. The interfaces between groups were accessed by one-way variance analysis followed by the Bonferroni post-hoc test for numeric variables. The Kruskal–Wallis test was used to analyze the histopathological score. The correlation levels were evaluated by the Pearson correlation coefficient (*r-value* < 0.7 for a strong correlation). The level of significance adopted was *p* ≤ 0.05. The data were described as the mean ± SEM. Statistical analyses were performed in the software PASW Statistics software version 22.0 (IBM Corp., Armonk, NY, USA). All other tasks were performed in the Microsoft Excel (2010) program (Microsoft, Albuquerque, NM, USA).

## 5. Conclusions

These pieces of evidence indicate that one week of a high-fat diet is enough to trigger pro-inflammatory mechanisms in peripheral key tissues. We also concluded that the juçara dose of 0.25% is more suitable to produce positive metabolic effects, prevent body weight gain, and liver injury. It becomes clear that food should be considered in its whole and not a single nutrient. Higher doses of supplementation did not represent a more pronounced or better effect on the inflammatory, metabolic, or histological parameters. Additionally, a 0.25% dose of juçara could be considered a tool for the treatment and prevention of metabolic damage associated with a high-fat diet in liver. Nevertheless, further investigations about the effect of this powerful fruit must proceed to clarify the effects.

## Figures and Tables

**Table 1:** Absolute tissue weight on different experimental groups.

	Absolute tissue weight (g)				
	Control (*n* = 6)	HFC (*n* = 7)	HFJ0.25% (*n* = 7)	HFJ0.5% (*n* = 7)	*p-*Vaule
Liver	10.57 ± 0.54	11.46 ± 0.66	10.23 ± 0.70	10.16 ± 0.85	-
RET	3.39 ± 0.52	6.99 ± 0.90 ^#^	4.67 ± 0.71	4.95 ± 0.64	0.029
EPI	3.74 ± 0.38	5.67 ± 0.69 ^#^	4.20 ± 0.61	3.94 ± 0.70	0.044
MES	3.57 ± 0.64	4.18 ± 0.44	3.44 ± 0.26	3.99 ± 0.50	-
ΣWAT	10.70 ± 1.36	16.84 ± 1.64 ^#^	12.32 ± 1.37	12.88 ± 1.76	0.020

# *p* < 0.05 vs. control group by two-way ANOVA followed by the Bonferroni post-hoc test. The *p*-value is shown in the table above. Control standard chow (control); high-fat diet control (HFC); high-fat diet juçara 0.25% (HFJ0.25%); high-fat diet juçara 0.5% (HFJ0.5%). RET, retroperitoneal tissue; EPI, epididymal white adipose tissue; MES, epididymal white adipose tissue; ΣWAT, sum of white adipose tissues’ weight depots.

**Table 2:** The histopathological score in liver among the groups (*p* > 0.05).

	0	1	2	3
Control (*n* = 5)	5	0	0	0
HFC (*n* = 7)	4	3	0	0
HFJ0.5% (*n* = 7)	3	4	0	0
HFJ0.25% (*n* = 7)	5	2	0	0

The Kruskal–Wallis test was used to analyze the histopathological score among the experimental groups. Control standard chow (control); high-fat diet control (HFC); high-fat diet juçara 0.25% (HFJ0.25%); high-fat diet juçara 0.5% (HFJ0.5%).

**Table 3:** Hepatic enzymes analyzed in serum and liver ectopic triacylglycerol storage.

	Control (*n* = 6)	HFC (*n* = 7)	HFJ0.25% (*n* = 7)	HFJ0.5% (*n* = 7)
AST (U/L)	36.18 ± 2.77	49.43 ± 4.99 ^&^	34.11 ± 7.25	49.79 ± 6.31 ^&^
ALT (U/L)	16.61 ± 1.37	23.06 ± 9.89	15.41 ± 3.21	17.17 ± 4.58
TAG (mg/100mg)	129.91 ± 4.41	157.33 ± 4.92 ^#^	142.59 ± 5.21	151.71 ± 2.14 ^#^

AST: aspartate transaminase; ALT: alanine transaminase; TAG: triacylglycerol. ^#^ *p* < 0.05 vs. the control group using two-way ANOVA followed by the Bonferroni post-hoc test; ^&^ *p* < 0.05 vs. the control group using the *t*-test.

**Table 4:** Composition of the experimental diets used: high-fat diet control, high-fat diet juçara 0.25%, and high-fat juçara 0.5%.

Components	Diet (g/100g)			
	Control	HFC	HFJ0.25%	HFJ0.5%
Standard chow *	100	50	50	50
Sucrose	-	10	10	10
Casein	-	20	20	20
Soybean oil	-	2	2	2
Lard		18	18	18
Butyl hydroquinone	-	0.004	0.004	0.004
Juçara pulp powder	-	-	0.25	0.5
Mineral mix^® §^		0.5	0.5	0.5
Vitamins mix^® &^		1.75	1.75	1.75
Energy (Kcal/100g)	270	410	420	430

* The standard rat commercial chow used was Nuvilab CR1 (Nuvital, Brazil); ^§^ mineral mix (AIN-93M, mineral mix, Rhoster, Brazil); ^&^ vitamin mix (AIN-93M, vitamin mix, Rhoster, Brazil).

**Table 5:** The main bioactive compounds and fatty acids detected in juçara pulp powder.

**Bioactive Compound**	**Concentration (in 100 g Dry Basis)**
Cyanidin 3-rutinoside (mg)	1790.0 ± 57.5
Cyanidin 3-glucoside (mg)	740.9 ± 22.1
Total anthocyanins (mg)	2663.7 ± 76.2
Apigenin deoxyhexosyl-hexoside (mg)	224.7 ± 13.2
Luteolin deoxyhexosyl-hexoside (mg)	332.7 ± 16.8
Dihydrokaempferol-hexoside (mg)	587.6 ± 23.0
Total phenolic compounds (mg)	3976.1 ± 197.34
Total fiber (g)	28.3 ± 0.3
**Fatty Acids**	**(% Fatty Acids Total)**
Oleic acid (C18:1)	44.2
Linoleic acid (C18:2)	17.4
Linolenic acid (C18:3)	0.45

**Table 6:** Score levels’ classification considering the area of necrosis and hepatic alterations previously established by Silva et al. (2014) [37].

Score	Necrosis Area	Alterations
0	0%	Preserved structures
1	<30%	Discreet eosinophilia, the presence of intracytoplasmic vacuoles
2	≥30%	Marked eosinophilia, dilation of sinusoid space, congested vessels, and intracytoplasmic vacuoles
3	≥50%	Marked eosinophilia, congested vessels, intracytoplasmic vacuoles, karyolysis, and structural loss
